# Empirical Performance Analysis of Hyperledger LTS for Small and Medium Enterprises

**DOI:** 10.3390/s22030915

**Published:** 2022-01-25

**Authors:** Dodo Khan, Low Tang Jung, Manzoor Ahmed Hashmani, Moke Kwai Cheong

**Affiliations:** 1 Department of Computer and Information Science, Universiti Teknologi PETRONAS (UTP), Seri Iskandar 32610, Malaysia; lowtanjung@utp.edu.my (L.T.J.); manzoor.hashmani@utp.edu.my (M.A.H.); kwai_17002385@utp.edu.my (M.K.C.); 2 High Performance Cloud Computing Center (HPC3), Universiti Teknologi PETRONAS (UTP), Seri Iskandar 32610, Malaysia

**Keywords:** blockchain, Hyperledger fabric, throughput, latency, scalability, workload variance

## Abstract

The massive success of blockchain technology in Bitcoin is taking the world by storm by attracting vast acceptance from both the public and private sectors. Blockchain allows digital transactions between two parties without a third party as a broker. Blockchain is now applicable beyond fintech to various other industries. Among these, Hyperledger fabric has emerged as the most popular blockchain-based open-source permissioned platform targeting business applications. It has been used in over 400 proofs-of-concept blockchain and is well proven in applications, such as supply chain, healthcare, and so on. Despite the many obvious benefits observed in blockchain-enhanced platforms, there still exist technical challenges in scalability, causing performance deficiency, which includes latency and throughput. There is an exigent need to improve the current blockchain-based applications to have the blockchain nodes be scalable without compromising the blockchain performance. In this study, we present the impact of workload variance of up to 1000 transactions with the setup of 20 blockchain nodes in the Hyperledger LTS platform. The evaluation metrics are transaction success and failure rate, throughput, and latency in the blockchain. The transaction throughput was found to be consistent with the increasing workload on a constant number of nodes. However, it showed a declining trend with an increasing number of nodes. As far as the latency, it was in tandem with the increased workload and the number of nodes. We, therefore, conclude that the LTS version is suitable for small and medium enterprises that do not scale up.

## 1. Introduction

The massive success of Bitcoin introduced by Satoshi Nakamoto in 2009 [1] has seen blockchain technology taking the world by storm. Since then, it has been attracting vast attention from the industry and academia. It is obvious that most of the cryptocurrencies in the market now are blockchain based and it would not be wrong to frame blockchain as the backbone of cryptocurrencies. Blockchain introduces a completely new approach for data storage, monitoring, and transaction. Blockchain allows digital transactions between two parties without a third party as a broker [2]. As a matter of fact, the applicability of this technology is beyond the financial sector, and it has already been implemented in many other non-financial sectors, such as smart cities [2,3], healthcare [4,5,6], IoT [7,8,9], supply chain [10], 5G networks [11], and many others [12].

Blockchain is a distributed and decentralized ledger of cryptographically signed transactions. Instead of a single entity, the entire network is managed and maintained in a trustless environment. Every node in the network carries a similar copy of data, which is stored in different blocks. These blocks collectively form a chain of blocks, where the blocks are connected in chronological order [13]. Besides being distributed in nature, blockchain allows transactions to be anonymously performed in the network between business partners without a centralized authority [14]. Instead, a cryptographic algorithm verifies the authenticity of the transaction.

Blockchain is generally categorized into two major categories: permissionless or permissioned. In the permissionless blockchain environment, a node is allowed to leave or join the network on its preferred choice and is allowed to perform a transaction. Bitcoin [1] and Ethereum [4] are examples of permissionless blockchain. Permissionless blockchain consists of a huge number of nodes, where a consensus mechanism, such as proof of work (POW) [15], is utilized to arrange transactions, then to verify and to create the blocks, whereas the permissioned blockchain is different in the way that nodes are known, identified, cryptographically authenticated, and the number of selected nodes are assigned to minimize the processing time [16] in the consensus process. Moreover, permissioned blockchain has strong, built-in access control to carefully define who is allowed to read a block, to append a block, to perform transactions, and to administer the participation in the blockchain network [13].

Since permissioned blockchain allows authenticated participation, it is, therefore, highly suitable for enterprise applications that require critical access control. There are several blockchain platforms available for permissioned/private settings, such as Ethereum [13,17], Hyperledger Fabric [18], and Corda. These platforms were developed to help enterprises, and businesses are utilizing blockchain in their respective applications. Hyperledger Fabric is the most accepted and popular platform and has been well proven in many enterprise applications, such as supply chain, healthcare [19], etc.

Despite the many obvious benefits of blockchain, there are still some technical challenges attached to it [20]. The major concern in adopting blockchain is its scalability and its performance related to scalability [21,22]. The comprehensive research in [23,24] proved that the major factors concerning performance with respect to scalability are latency and throughput [14]. Performance related to scalability is the main problem and it is hindering the wide adoption of blockchain in small and medium enterprises. The small and medium enterprise applications will inevitably evolve over time to run over a large number of nodes (scaling up) [24]. There is, therefore, an exigent need to improve the current blockchain-based applications to make blockchain nodes scalable without compromising performance. There exists the need to enhance or to provide practical options to replace the current systems [21]. Moreover, there is no reference in the literature that guides small and medium enterprises (SME) to select suitable blockchain platforms for their respective business in considering the scaling up of blockchain in coming years.

This paper is about our empirical performance analysis on one of the well-known blockchain platforms—the Hyperledger Fabric LTS version aiming for the small and medium enterprise (SME) domain. The results of this analysis can serve as references for SMEs to understand the performance of this platform when they integrate it with their respective real-world applications. The knowledge gained regarding its limitations will eventually assist SMEs in selecting the platform to suit a particular business. Furthermore, Hyperledger Fabric’s fast adoption in many businesses has demanded some critical performance assessment. It is, therefore, highly important to analyze the throughput, the transaction rejection probability, and the mean transaction response delay so that providers can select the best platform that maximizes their profits in a respective business.

Hyperledger Caliper [25,26] was utilized as the main benchmarking tool in our empirical performance analysis. Our analysis includes the success rate, throughput, and average latency. The analysis mainly focused on the setup involving varying the number of transactions (workload) and the number of clients (nodes). The main contributions of this work are summarized below:

- The impact of system configurations (total number of transactions, number of nodes) on the performance with respect to blockchain scalability.
- The performance analysis results as a practical reference for SMEs practitioners in selecting the Hyperledger Fabric LTS version for their business applications.

The structure of this paper is as follows. Section 1 introduces this article. Section 2 discusses the background of Hyperledger with respect to blockchain technology. Section 3 and Section 4 present the related work and the research methodology of this study. Section 5 discusses the results and findings. Section 6 concludes this paper.

## 2. Background

Hyperledger is a collection of free and open-source projects and applications maintained by the Linux Foundation established in December 2015. This initiative is divided into five subprojects, namely, Hyperledger Iroha [27], Hyperledger Sawtooth [28], Hyperledger Fabric [11], Hyperledger Indy [29], and Hyperledger Burrow [30]. This paper covers only Hyperledger Fabric. Hyperledger Fabric is the most common among the open-source permissioned blockchain frameworks [13] targeting the business applications [31]. It does not support cryptocurrencies, such as Bitcoin or Ethereum. Entry to the network is limited to permitted network users only. It is currently being used in over 400 proofs of concept and production distributed ledger applications across a variety of sectors and use cases [32].

Hyperledger Fabric (HLF) is based on private (permissioned) blockchain technology. Its architecture is designed to serve as a framework for building blockchain applications across a broad range of industries [33]. The architecture is modular and allows components, such as consensus and membership services, to be added and removed as required. It encompasses the container approach (docker) to allow smart contracts, known as chaincode, to form the system’s application logic [34]. The privacy of the transactions in the network is achieved by incorporating an isolation system known as a channel to ensure that only the authorized nodes of a particular channel can access the transaction. The main components in HLF are presented in the sub-sections below. To run HLF, it is recommended to have a computing system with at least 4 GB of memory running on operating systems such as Ubuntu Linux 14.04/16.04 LTS (both 64-bit), or Mac OS 10.12. Other hardware requirements are entirely dependent on the number of Org, peers, and channels. However, many implementations used 2 CPUs (minimum 1), 4 GB RAM, and 30 GB disk space.

### 2.1. Membership Service Provider (MSP)

The membership service provider (MSP) specifies the rules for validating and authenticating identities and granting entry to a blockchain network. The MSP manages user IDs and authenticates clients who wish to access the network. This includes providing certificates to these clients for them to propose transactions. The MSP utilizes certificate authority (basically a plug and play interface) which verifies or revokes the user certificate based on the identity verified.

### 2.2. Client

The client system is for creating a transaction proposal. The client proposes the transaction request to many peers at the same time to gather proposal responses of the endorsements to fulfill the endorsement policy. The transaction is then broadcasted to various orderer nodes to be used in a block, and that block is shared with all peers for verification and commitment.

### 2.3. Peer

Peer nodes are used to run the chaincode, which implements user-based smart contracts and stores the ledger in a file system. Well-defined ledger APIs grant chaincode access to the shared state. A peer is further classified as either an endorsing peer or committing peer. The endorsing peer has the chaincode logic and uses this logic to endorse a transaction. The committing peer does not have the chaincode logic. Regardless of this distinction, all groups of peers keep the ledger. Furthermore, all endorsing peers and the committing peers keep the current state as StateDB in a key–value store, allowing the chaincode to access or change it, using a database query language.

### 2.4. Channel

**Figure 1:**
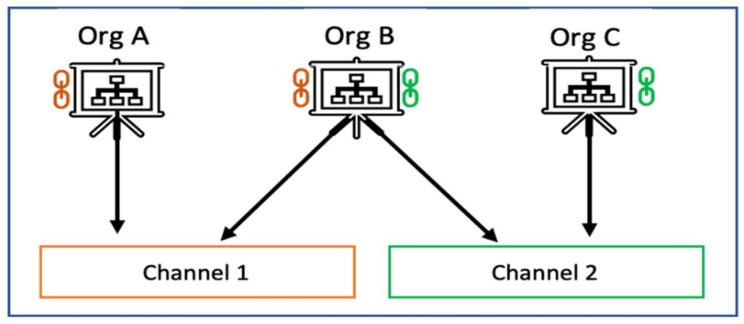
Channel structure. Source: [12].

HLF supports the existence of several channels within the same network based on the requirements. A channel is a private communication system for entering into the larger network. Every channel has its own ledger (all organizations and peers who are part of that channel have a copy of the ledger). The channel members are stated in the channel policy, which is part of the configuration block that also specifies the type of ordering service. Any alteration in the configuration block generates a configuration block and becomes part of the main chain. The channel concept is shown in Figure 1. Here, organizations A and B are part of Channel 1, and organizations B and C are part of Channel 2. The peers in the organizations in Channel 1 possess a copy of the ledger that is only relevant to Channel 1. As such, peers in organization C possess a copy of the ledger that is exclusive to Channel 2. Organization B’s peers have a copy of both ledgers and, therefore, are members of both networks. This may be helpful in situations when involving competing companies, as it enables private contact between the organizations to take place only on the same channel.

### 2.5. Orderer

HLF implements orderer nodes to achieve consensus. The primary duty of an orderer node, as the name suggests, is to order transactions into the block and to broadcast that block to all peers for confirmation of the transaction. HLF has three separate ordering service implementations. The ordering service is modular and has a configurable consensus system.

#### 2.5.1. Solo

There is only one ordering node in a solo-based implementation. It, therefore, has a single point of failure. As a result, a solo-based ordering service is unsuitable for production. Nonetheless, since it removes the operating overhead associated with most deployment systems, it may be used for testing and scholarly purposes.

#### 2.5.2. Kafka

This model employs a simple strategy of follower and a leader. The leader orderer is responsible for sending transactions to the follower orderer nodes. The selection of leader is done dynamically as long as the rest of the nodes are operational. This system is crash fault tolerant (CFT). For handling Kafka clusters, this model makes use of the zookeeper coordination service. Zookeeper is a service utilized by distributed applications to hold configuration information, assist in task management, provide distributed synchronization, and cluster participation. Although this scheme is the only solution that supports multiple orderers since HLF v1.0, setting up a Kafka-based ordering service is difficult and requires expert deployment.

#### 2.5.3. Raft

HLF recently introduced the new ordering service called Raft, which is built on the Raft protocol. It is like Kafka in that it is CFT and uses the leader-and-follower strategy. There are not many technical differences between Raft and Kafka. Raft is, however, easier to set up as compared to Kafta.

### 2.6. Chaincode

The business logic into the transactions is actually defined by smart contracts, which particularly govern the business entity life cycle, and is embedded in the world state which is packaged into chaincode. It is then distributed to the entire blockchain network. Therefore, smart contracts are defined by chaincode. A single chaincode allows the specifying of multiple smart contracts into it. After the implementation of the chaincode, the application can use all the available smart contracts within that chaincode. Chaincode is technically a container for installing and instantiating several similar smart contracts, whereas smart contracts are specific to the application that is applied to allow business processes. Every chaincode comes with an endorsement policy that extends to each relevant smart contract linked to it. This specifies which organization must sign a smart contract-generated transaction for it to be considered a valid organization. In brief, a smart contract is accompanied by an endorsement strategy. A smart contract can communicate with other smart contracts on the same or a different channel.

### 2.7. Transaction Flow in Hyperledger Fabric

**Figure 2:**
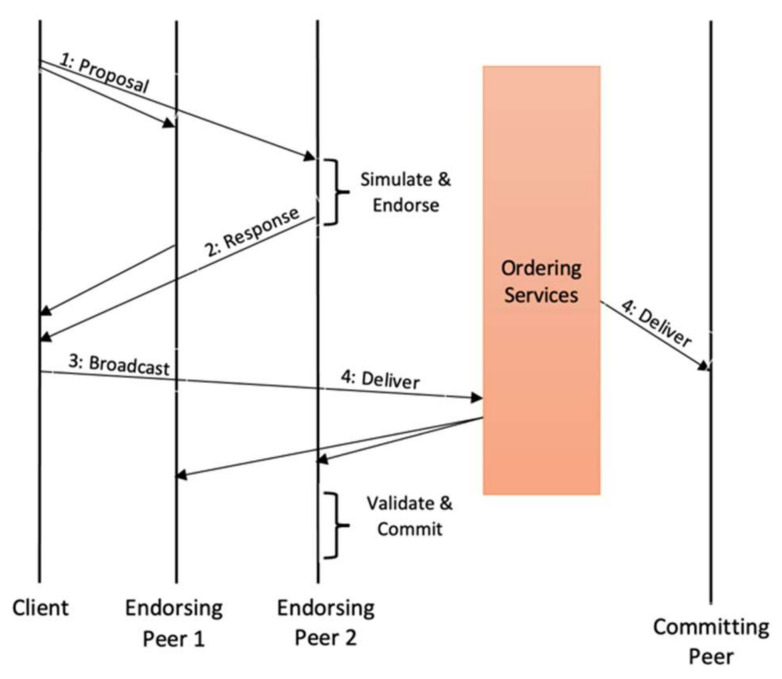
Transaction flow of ordering service. Source: [13].

In the Hyperledger Fabric blockchain, a successful transaction goes through three stages, which are execute, order, and validate. These stages are illustrated in Figure 2. The process begins with the transaction proposal and concludes when the transaction is committed to the ledge. Firstly, the proposed transaction goes through 2 initial stages, which are execute and endorse. Secondly, by a consensus system, the ordering service orders these transactions into a block. Finally, peers validate the contract to avoid conflicts caused by concurrency.

#### 2.7.1. Phase 1: Proposal (Execute)

The main goal of this process is to endorse the proposed transactions. The initial phase begins when a Fabric SDK-enabled program generates a transaction proposal to the endorsing peers. The endorsement strategy determines the group of endorsing peers to be selected. All peers perform the following verifications:

- The transaction proposal is structured correctly.
- It is not duplicating an already existing transaction.
- The issuer’s signature is valid.
- The transaction issuer is permitted to execute the proposed operation.

Then, chaincode is executed by each endorser one at a time and produces a response of transaction which entirely depends on the execution results before signing the response. Finally, the application receives the signed transaction proposal response. The client does not end the first step until it obtains a certain number of endorsements based on the number of endorsing peers specified in the endorsement policy.

#### 2.7.2. Phase 2: Ordering and Packaging (Order)

Clients are responsible for generating transactions and broadcasting all the transactions to the ordering service. A normal transaction consists of a list of endorsements, transaction metadata, and payload, along with the channel ID. First, the orderer receives transaction proposal responses from the clients. Then, the orderer packages the approved transactions into a block in sequential order. The ordering service is not allowed to read the contents of the transactions. However, it maintains consensus and a complete order on all transactions per channel while utilizing a plug-and-play consensus protocol after which the ordering service manages the ordered transactions into a block and distributes the blocks to peers, using the gossip protocol.

#### 2.7.3. Phase 3: Validation

Both peers, including endorsers and committers, collect the block from the ordering service and decode it. All peers on the channel are allowed to verify the transactions in the block independently. All peers validate the block in exactly a similar method. Therefore, every peer holds a similar copy of the ledger. All peers check the orderer’s signature on the block, then all the checked signatures are decoded, and all transactions are validated using the validation system chaincode (VSCC) before moving on to multi version concurrency control (MVCC).

- VSCC validation: A validation system chaincode is responsible for comparing the list of endorsements in the transactions with the endorsement policy (listed for the chaincode). If it is noted that the endorsing policy is not followed during the process, then that transaction is declared invalid.
- MVCC validation: MVCC is also known as a read–write dispute search because it guarantees that the versions of keys read or written during the execution process match the actual ledger state. This MVCC is applied sequentially to all transactions in a block. Transactions are flagged as invalid if the versions do not align.

Ledger update phase—when the MVCC and VSCC validations are complete, all peers commit blocks and add them to the locally stored ledger as the final stage in transaction processing.

## 3. Related Work

In this section, the recently published research on the evaluation of the performance of Hyperledger Fabric is discussed. The past five-year research works are considered in this section.

Xiaoqiong Xu [35] stated that the latency efficiency of the Hyperledger Fabric blockchain is critical in determining its efficacy. He suggested a novel framework to measure the transaction latency under unlikely network settings, such as block interval and block size. The findings revealed that there is a 6.1 percent variance (difference) between the analytical and experimental results. Another research work in [36] utilized Hyperledger in the healthcare domain and designed a blockchain-based low-cost multi-platform application, which benefits all stakeholders in the healthcare industry (patients, hospitals, pharmaceuticals, and health insurance).

Lili Jiang [28] considered two critical factors: ignored block timeout and transaction endorsement failure. These were previously overlooked in the efficiency assessment for successful transaction processing. He introduced a hierarchical model for the transaction mechanism in Hyperledger Fabric v1.4. Formulae for measuring the performance metrics, such as platform throughput, transaction rejection probability, and mean transaction response latency, were appropriately derived. He used simulations to validate and approximate the accuracy of the model and the formulae derived.

Arati Baliga [29] assessed Quorum’s performance. The throughput and latency of various workloads and consensus algorithms were the main considerations. The micro-benchmarks were used to determine how different transaction and smart contract parameters impacted the transaction latencies.

Canhui Wang [30] performed a detailed performance assessment of Hyperledger Fabric in accordance with the new architecture. Each process, including the execute, request, and validate phases, as well as all pluggable ordering services, such as Solo, Kafka, and Raft, were thoroughly analyzed. His experimental findings revealed that the scalability of execution phases was determined by the OR and AND endorsement policies, and no substantial output discrepancy between the three ordering services was observed.

The recent study in [27] evaluated Ethereum, Parity, and Hyperledger Fabric in depth. The findings showed that there were performance differences between the three systems, but none of them came close to displaying a performance that is comparable to the existing database systems in the conventional data-processing workloads. BLOCKBENCH, the first assessment method, was used in the analysis.

The research in [7] evaluated the HLF by applying and benchmarking a digital currency HPL to produce a throughput of higher than 3500 TPS in some common implementation setups with sub-second latency. The scaling went well to more than 100 peers as reported in this article.

Qassim Nasir [37] compared the results of the two HLFs—versions 0.6 and 1.0 by varying the workloads with key measurements based on latency, execution time, and throughput. The scalability was assessed by varying the number of nodes in the blockchain network. The results revealed that Hyperledger Fabric v1.0 reliably outperformed v0.6 in terms of scalability, throughput, execution time, and latency. The performance of HLF v1.0, however, did not match well with the existing standard database systems under high workload conditions.

Supornn Pongnumkul [34] compared the performance of the Ethereum and Hyperledger Fabric networks with differing transaction volumes. As workloads varied up to 10,000 transactions, the results showed that Hyperledger Fabric achieved higher throughput and less latency than Ethereum. The results further showed that as the transaction grows, the variations in execution time and the average latency between these two platforms became more pronounced. However, with comparable computing capital, Ethereum can accommodate a greater number of concurrent transactions.

Harish Sukhwani [38] explored the impact of a consensus mechanism based on PBFT on peer evaluation performance with a wide number of peers. When running an IoT system, Harish used a PBFT consensus approach in stochastic reward nets to measure the consensus meantime for networks up to 100 peers. The data obtained were then used to parameterize and verify and validate the proposed models.

Yue Hao [39] investigated the impact of consensus protocol in HLF performance evaluation. A novel method was proposed to evaluate the performance of consensus algorithms in permissioned blockchain networks, such as HLF and Ethereum. By conducting a comparative study on the throughput and the latency results, the consensus mechanism seemed to have created an efficiency bottleneck. It was observed that the PBFT consensus model consistently outperformed proof of work in terms of throughput and latency under variable workloads.

Parth Thakkar [13] conducted a thorough analysis to describe the efficiency of Hyperledger Fabric. The analysis was conducted based on the effect of various configurations such as endorsement policy, block size, resource allocation, latency, and state database preference on transaction throughput networks. The analysis provides various recommendations on configuring these parameters. The three main bottlenecks were found to be the state validation and commit (with CouchDB), the sequential policy validation of transactions in a block, and the endorsement policy authentication.

Murat Kuzlu [14] examined the effect of network workload on the scalability, transaction throughput, reliability, and transaction latency of the HLF. The results highlighted that the HLF network could accommodate up to 100,000 participants on the chosen AWS EC2 event. If the transaction rate was held under 200 TPS, the latency of the network was in the order of fractions of a second.

Salma Shalaby [12] explored the possibilities of customizing the blockchain network to the needs of the applications. Several studies were conducted to assess the efficiency of the HLF. Seven separate scenarios were evaluated on blockchain in terms of end-to-end transaction latency and network throughput. Furthermore, the effect of various criteria, such as batch-timeout, batch duration, and several endorsing peers, was also analyzed in those seven scenarios.

Mohammad Dabbagh [21] carried out an observational analysis to compare the performance of HLF and Ethereum with respect to four metrics: average latency, performance rate, resource utilization, and throughput. The analysis results based on 100 transactions revealed that HLF outperformed Ethereum in all four metrics. 

Table 1 presents a comparative analysis of currently available research on the evaluation of the performance of Hyperledger Fabric in contrast to this study. 

## 4. Methodology

### 4.1. Experiments

Based on the Hyperledger Fabric white paper, two experiments were crafted to evaluate the performance of the Hyperledger Fabric.

- Experiment 1: To evaluate the performance by having the workload as a variable. This includes the number of transactions and the simultaneous requests by the same number of nodes.
- Experiment 2: Evaluate the scalability by having the number of nodes as a variable. The threshold was set to 20 nodes, with the same/constant workload.

### 4.2. Application: Simulated Application and Smart Contracts

A simple car-buying application was developed for the evaluation of the performance of Hyperledger Fabric. In the application, there were two different functions (smart contract “Create” and “Query”) developed to generate a car and to generate a query on the generated cars.

### 4.3. Hyperledger Fabric Deployment/Blockchain Platforms

**Figure 3:**
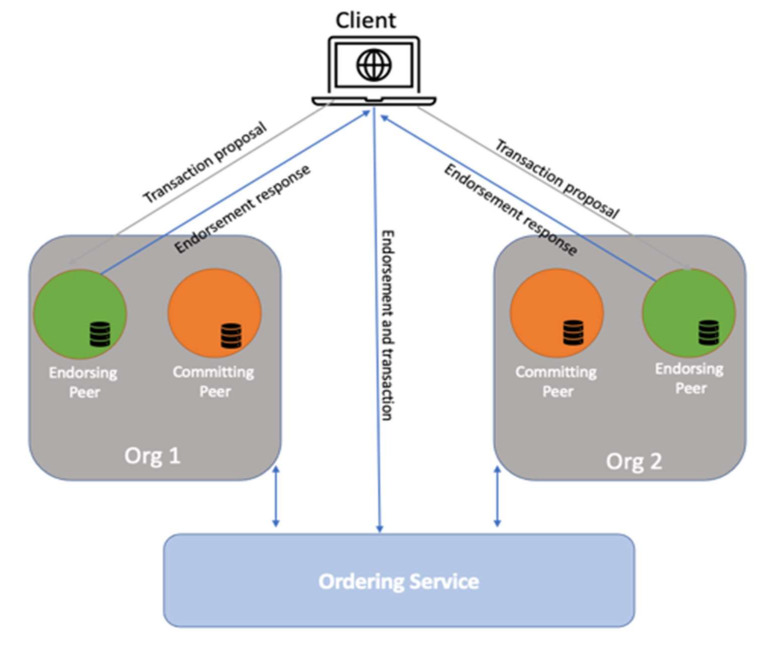
Illustrating the major blockchain components.

Hyperledger Fabric was deployed independently. All relevant components were installed, which included two organizations (Org1 and Org2) with four peers: two peers (1 committing peer and 1 endorsing peer) per organization along with four CouchDB instances. The RAFT consensus mechanism was deployed with the ordering service. The implemented model is conceptually represented in Figure 3, illustrating the major blockchain components together with all used tools. In our study, we focused on evaluating the performance of the HLF LTS version. The experiments were performed on the Intel(R) Xeon(R), 2.6 GHz with 12 core CPU, 16 GB RAM, 500 GB disk space, and running on Ubuntu 18.04 LTS.

The Hyperledger Fabric supports Java and JavaScript programming languages. Therefore, in this study, the smart contracts were written in GO, and the Hyperledger Fabric Client SDK Node.js was used for interacting with Hyperledger Fabric.

### 4.4. Hyperledger Benchmark Tool

Hyperledger Caliper (2019) was used to generate the workload for Hyperledger Fabric. The caliper is a performance benchmarking tool commonly used to measure and evaluate the performance of blockchain applications. The caliper can generate the performance report through customized use cases. The report can include performance indicators, such as the success rate, throughput, latency, and resources utilization. In this study, the caliper executes all client nodes where a measured workload with a defined sending rate is sent by each client. For setting up the benchmark tool, a configuration file was needed to update the transaction rates, data, and data types.

### 4.5. Evaluation Metrics:

In our empirical analysis, the focus was the main primary metrics of the HLF setup: success rate, transaction throughput, and transaction latency. Each of these is explained below.

- Throughput is described as the number of successful transactions per second.
- Average latency is specified as the average time interval between the initialization of the transaction and the actual execution of the transaction.
- The success rate of a blockchain is determined by the number of successful transactions performed out of the total transactions.

## 5. Results and Discussion

The evaluation process was based on the two experiments mentioned above, that is, the performance assessment on two peers and the scalability efficiency of the HLF blockchain network. The results were measured using Hyperledger Caliper. The empirical analysis is presented below.

### 5.1. Performance Assessment

**Figure 4:**
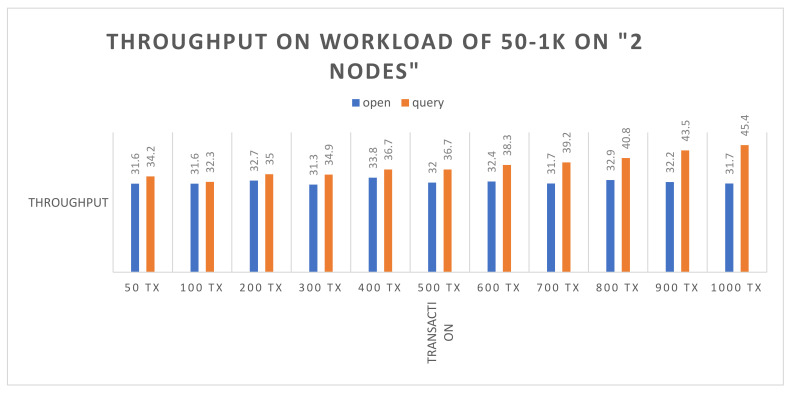
Transaction throughput on the open and the query functions.

**Figure 5:**
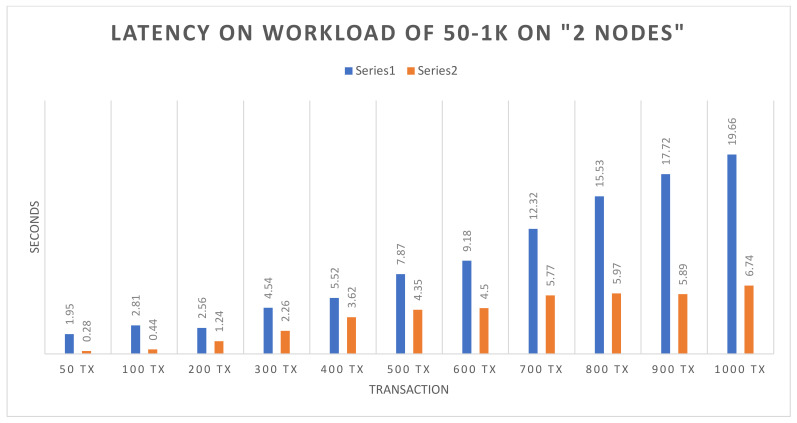
Average latency on the open and the query functions.

- Success rate: The experiment evaluation reveals that both the (open and query) functions attained a 100% success on 50–1000 simultaneous transactions on same number of nodes.
- Throughput: Figure 4 illustrates the transaction throughput after executing the open and query functions using 50 to 1000 simultaneous transactions. The blue bars show the open function, and brown bars highlight the query function. It is observed that the throughput on the query function is slightly higher than the open function. Initially, the throughputs on both functions are almost equal. As the number of transactions increases up to 600, there is slight growth observed in the throughput in the query function, whereas the open function shows consistency in the throughput. The continuous consistency observed in the throughput reflects the reliability and availability of Hyperledger. In the following figure, the *X*-axis represents the number of transactions, and the *Y*-axis represents the throughput.
- Average latency: Figure 5 shows the average latency after executing the open and the query functions, using 50 to 1000 simultaneous transactions. The blue bars show the open function, and brown bars highlight the query function. It is noticed that there is continuous growth in the average latency as the number of transactions are increasing for both the query and the open functions. However, the query function continuously has more latency than the open function, and it achieves more growth after 600 transactions. However, the average latency of the query function is higher than the open function. In the following figure, the *X*-axis represents the number of transactions, and the *Y*-axis represents the seconds.

### 5.2. Scalability Assessment

The scalability of the Hyperledger Fabric LTS version was analyzed by varying the number of nodes up to 20. The metrics to assess scalability include the success rate, throughput, and average latency. The scalability was evaluated with 500 and 1000 transaction workloads.

**Figure 6:**
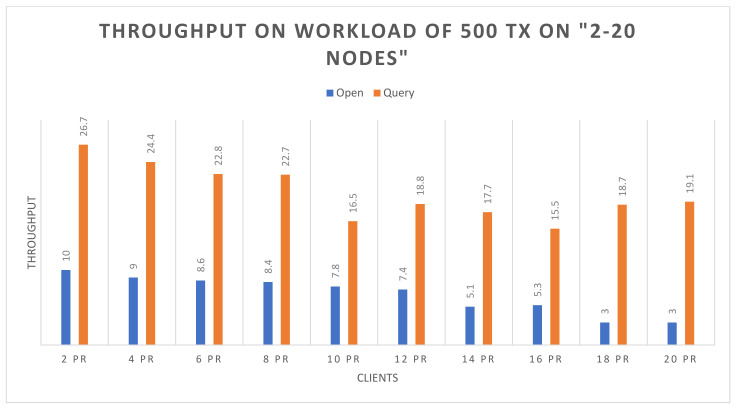
Throughput on 500 transactions.

**Figure 7:**
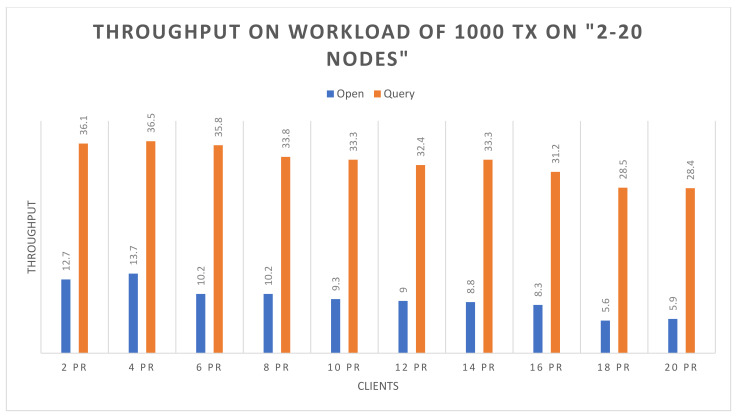
Throughput on 1000 transactions.

**Figure 8:**
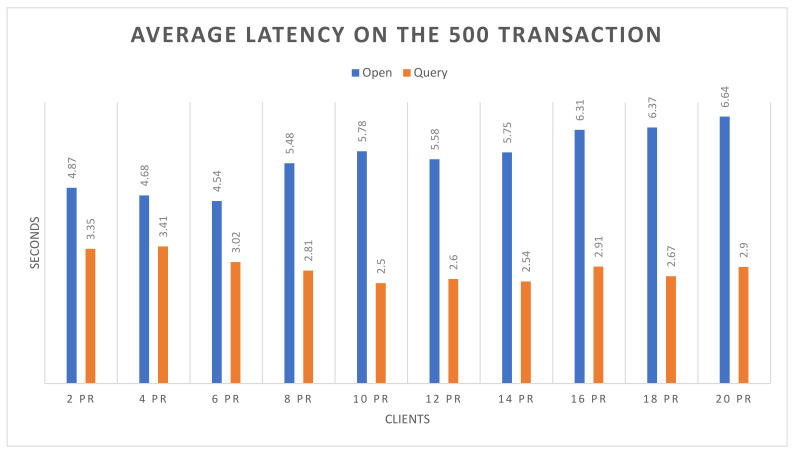
Average latency on 500 transactions.

**Figure 9:**
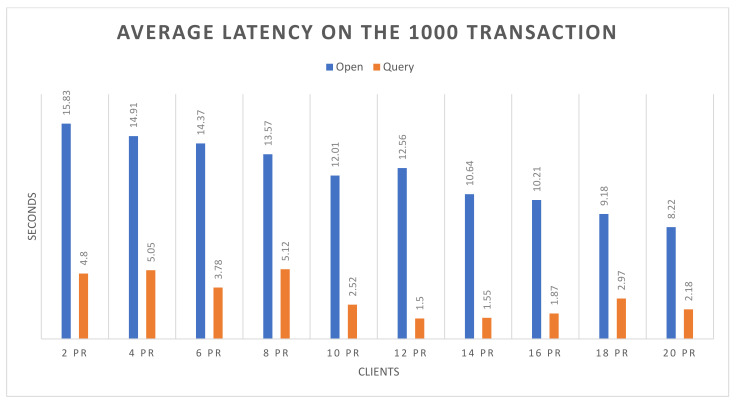
Average latency on 1000 transactions.

- Success rate: The experimental analysis reveals that both the open and query functions attended 100% success up to 20 nodes, with respect to 500 and 1000 transactions.
- Throughput: Figure 6 and Figure 7 demonstrate the throughput for executing the open and the query functions up to 20 nodes, based on 500 and 1000 transactions respectively. The blue bars show the open function, and brown bars highlight the query function. In Figure 6, it can be observed that the query function obtains higher throughput than the open function. However, the throughput of the query function decreases with the increasing number of nodes, and it also slightly decreases in the open function. In Figure 7, on 1000 transactions, the throughout in the query function is higher than the open function, but there is a continuous increase in throughput on query function as the number of nodes increases. Although the open function obtains lower throughput, it shows consistency. In the following figures, the *X*-axis represents the number of nodes, and the *Y*-axis represents the throughput.
- Average latency: Figure 8 and Figure 9 demonstrate the average latency on executing the open and query functions on the 20 nodes, using 500 and 1000 transactions, respectively. The blue bars show the open function, and the brown bars highlight the query function. In Figure 8, overall, the query function obtains lower latency than the open function, and as the number of nodes increases, the latency on both of the functions remains consistent. However, there is slight growth observed after the nodes are more than 10, whereas in Figure 9, initially the latency of the open function is quite high but as the number of nodes increases, the latency decreases, and the query function has more latency than the open function. However, after crossing the 8 nodes, there is a bit of consistency noticed in the query function. Overall, there is consistency noticed in the latency on the open function. In the following figure, the *X*-axis represents the number of nodes and the *Y*-axis represents the time in seconds.

## 6. Conclusions

This paper presents an empirical analysis on the performance of the Hyperledger Fabric LTS version as a permissioned blockchain platform. The analysis focused on varying the workload (transaction and request) and the number of nodes in the blockchain network. The impact on varying the workload up to 1000 transactions and scaling up to the 20 nodes were studied. The evaluation metrics used include success and fail, throughput, and average latency. The transaction throughput shows a bit of consistency with the increasing workload as well as in the increasing the number of nodes up to 20. However, the throughput decreases and remains inconsistent when the workload increases up to 1000 transactions on 20 nodes. As far as the latency is concerned, it increases with the increasing workload and in the number of nodes. However, it decreases on 20 nodes with 1000 transactions. We, therefore, conclude that this version of the Hyperledger is suitable for small and medium enterprises, and it would not be able to scale up to a high number of nodes and a high transactional rate.

## 7. Future Work

We have a plan to evaluate multiple consensus protocols with a high workload (transaction) and nodes in private blockchain networks. We shall also investigate the efficiency gaps between private and public blockchain systems with respect to SMEs blockchain applications.

## Figures and Tables

**Table 1:** Some recent experimental analysis of HLF and comparisons.

Article	Year	Cite	Title	Comments
[35]	2021	44	Latency performance modeling and analysis for Hyperledger Fabric blockchain network.	Focused on latency of Hyperledger Fabric. It also proposed a new framework to measure the latency.
[28]	2020	12	Performance analysis of Hyperledger Fabric platform: A hierarchical model approach.	Concentrated on two important factors: ignored block timeout and transaction endorsement failure. It also introduced a hierarchical model for the transaction mechanism in Hyperledger Fabric v1.4.
[29]	2018	38	Performance evaluation of the quorum blockchain platform.	Analyzed Quorum’s performance analysis. The throughput and latency of various workloads and consensus algorithms were taken into consideration.
[30]	2020	5	Performance characterization and bottleneck analysis of Hyperledger Fabric.	A thorough performance assessment of Hyperledger Fabric in accordance with the new architecture. Each process was assessed with respect to the execute, request, and validate phases.
[27]	2017	644	Blockbench: A framework for analyzing private blockchains.	Evaluated performance of 3 major platforms (Ethereum, Parity, and Hyperledger Fabric). Results showed that none of them came close in displaying performance comparable to the existing database systems.
[7]	2016	3203	Blockchains and smart contracts for the internet of things.	Evaluated the HLF by applying and benchmarking a digital currency HPL to produce a higher throughput in some common implementation setups with sub-second latency.
[38]	2017	245	Performance modeling of PBFT consensus process for permissioned blockchain network (Hyperledger Fabric).	Explored the impact of a consensus mechanism based on PBFT on peer evaluation performance with a wide number of peers when running an IoT system.
[39]	2018	70	Performance analysis of consensus algorithm in private blockchain.	Investigated the impact of consensus protocol in HLF performance evaluation. Proposed a novel method to evaluate the performance of consensus algorithms in the permissioned blockchain.
[12]	2020	12	Performance evaluation of Hyperledger Fabric.	Investigated the possibilities of customizing the blockchain networks for the needs of the applications.
This study	2021	-	Empirical performance analysis of Hyperledger LTS for small and medium enterprises.	Performed the analysis of Hyperledger LTS version, focusing on the real-world implementation of blockchain (HLF). Considered 3 critical metrics (success and fail rate, throughout, and latency) for SMEs businesses to select HLF. To serve as reference for SMEs to select suitable blockchain platform for their respective business in considering the scale up demands in coming years which are missing in all above articles.

## Data Availability

Not applicable.
